# Supplementary material for: Molecular definition of distinct active zone protein machineries for Ca2+ channel clustering and synaptic vesicle priming
Source: bioRxiv. 2023 Oct 30:2023.10.27.564439. Preprint. [Version 1] doi: 10.1101/2023.10.27.564439 (PMC10634917; doi:10.1101/2023.10.27.564439)
Supplement: Supplement 1 [file NIHPP2023.10.27.564439V1-supplement-1.pdf]

bioRxiv preprint doi: https://doi.org/10.1101/2023.10.27.564439; this version posted October 30, 2023. The copyright holder for this preprint (which was not certified by peer review) is the author/funder, who has granted bioRxiv a license to display the preprint in perpetuity. It is made available under aCC-BY 4.0 International license.

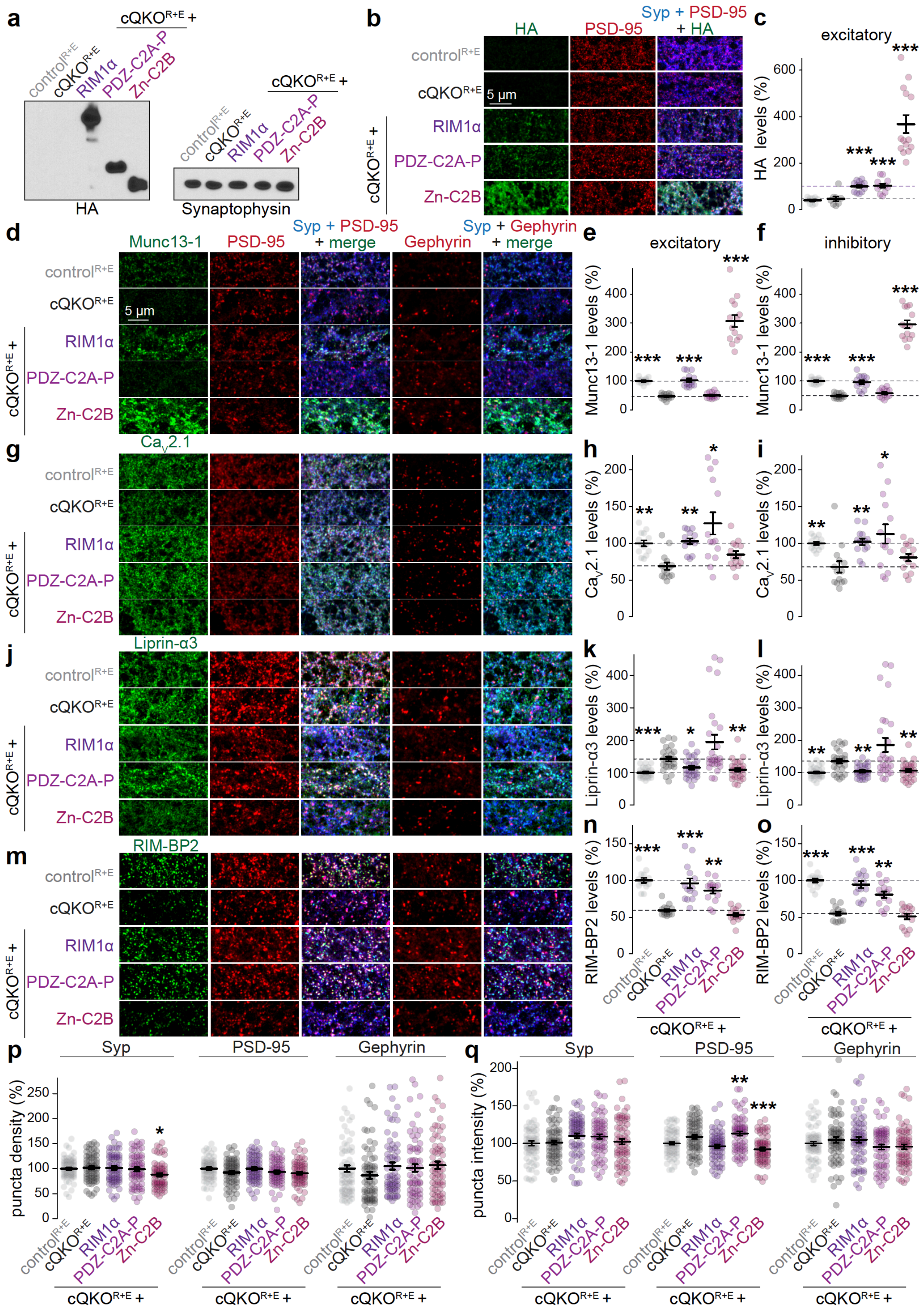

Supplemental figure 1. Assessment of RIM1 constructs with Western blot and confocal microscopy

(**a**) Western blot to assess expression of RIM1α, PDZ-C2A-P and Zn-C2B in cultured neurons.

bioRxiv preprint doi: https://doi.org/10.1101/2023.10.27.564439; this version posted October 30, 2023. The copyright holder for this preprint (which was not certified by peer review) is the author/funder, who has granted bioRxiv a license to display the preprint in perpetuity. It is made available under aCC-BY 4.0 International license.

(**b, c**) Example confocal images (b) and quantification (c) of the average intensity of HA (to detect rescue proteins) at excitatory synapses identified as PSD-95 regions of interest (ROIs). Neurons were stained for HA, PSD-95 and Synaptophysin (Syp). Intensity is normalized to the average $cQKO^{R+E}$ + RIM1α per culture. Levels at inhibitory synapses were not assessed due to incompatibility of HA and Gephyrin antibodies; $control^{R+E}$ 9 images/3 independent cultures, $cQKO^{R+E}$ 8/3, $cQKO^{R+E}$ + RIM1α 13/3, $cQKO^{R+E}$ + PDZ-C2A-P 12/3, $cQKO^{R+E}$ + Zn-C2B 14/3.

(**d-o**) Example confocal images and quantification of the average fluorescence intensity levels at excitatory and inhibitory synapses of Munc13-1 (d-f), $Ca_V2.1$ (g-i), Liprin-α3 (j-l) and RIM-BP2 (m-o). Neurons were stained for a protein of interest (Munc13-1, $Ca_V2.1$, Liprin-α3 or RIM-BP2), postsynaptic markers (PSD-95 and Gephyrin), and Synaptophysin. Excitatory synapses were defined as PSD-95 ROIs and inhibitory synapses as Gephyrin ROIs. Data are normalized to the average $control^{R+E}$ per culture, dotted lines mark the levels of $cQKO^{R+E}$ (black) or $control^{R+E}$ (gray); d-f, control 14/3, $cQKO^{R+E}$ 14/3, $cQKO^{R+E}$ + RIM1α 14/3, $cQKO^{R+E}$ + PDZ-C2A-P 13/3, $cQKO^{R+E}$ + Zn-C2B 14/3; g-i, 14/3 each; j-l, 26/6 each; m-o, 14/3 each.

(**p, q**) Quantification of Synaptophysin, PSD-95 and Gephyrin puncta densities (p) and of their fluorescence intensities (q) normalized to the average $control^{R+E}$ per culture. Small changes in Synaptophysin and PSD-95 in some conditions do not confound the conclusion that independent assembly pathways recruit Munc13-1 and $Ca_V2.1$; $control^{R+E}$ 68/6, $cQKO^{R+E}$ 68/6, $cQKO^{R+E}$ + RIM1α 68/6, $cQKO^{R+E}$ + PDZ-C2A-P 67/6, $cQKO^{R+E}$ + Zn-C2B 68/6.

Data are mean ± SEM; *$p < 0.05$, **$p < 0.01$, ***$p < 0.001$ compared to $cQKO^{R+E}$ as determined by Kruskal-Wallis followed by Holm multiple comparisons post hoc tests in c, e, f, h, i, k, l, n, o, p (Synaptophysin and Gephyrin) and q, or by a one-way ANOVA followed by Tukey-Kramer multiple comparisons post hoc tests in p (PSD-95).

bioRxiv preprint doi: https://doi.org/10.1101/2023.10.27.564439; this version posted October 30, 2023. The copyright holder for this preprint (which was not certified by peer review) is the author/funder, who has granted bioRxiv a license to display the preprint in perpetuity. It is made available under aCC-BY 4.0 International license.

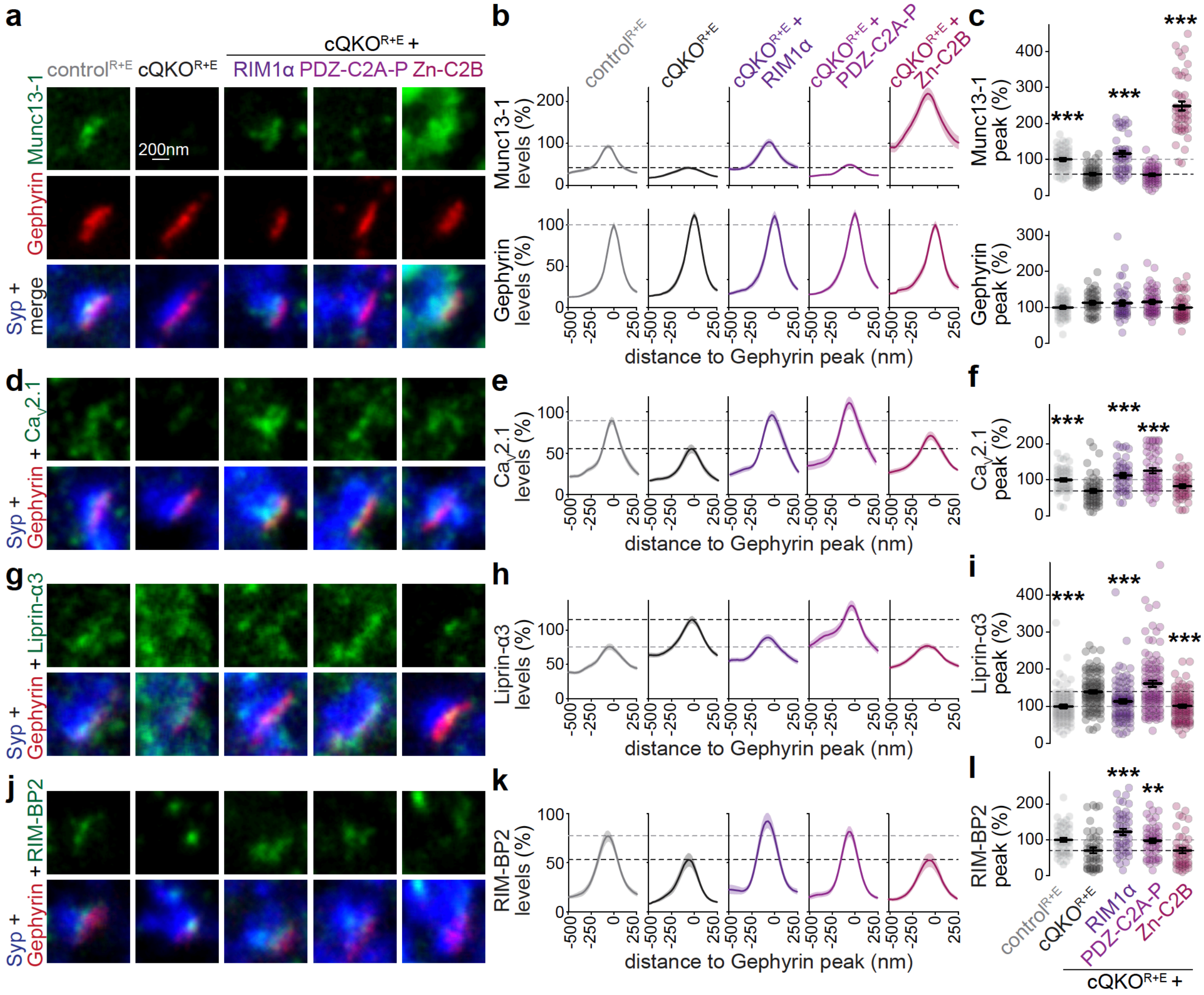

Supplemental figure 2. Assessment of inhibitory synapses by STED microscopy after RIM rescue

(**a-l**) Example STED images, average line profiles and quantification of the peak intensity of Munc13-1 and Gephyrin (a-c), $Ca_V2.1$ (d-f), Liprin-α3 (g-i) and RIM-BP2 (j-l) at inhibitory side-view synapses identified by Synaptophysin (Syp) and Gephyrin. Analyses were performed on the experiment shown in Fig. 2f-q as the neurons were co-stained for Gephyrin. A line profile (750 nm x 250 nm) was positioned perpendicular to the center of the elongated Gephyrin object and profiles of all synapses were aligned to the Gephyrin peak. The maximum value of each individual profile was used to calculate the peak. Dotted lines mark the levels of $cQKO^{R+E}$ (black) or $control^{R+E}$ (gray), line profiles and peak intensities are normalized to the average

bioRxiv preprint doi: https://doi.org/10.1101/2023.10.27.564439; this version posted October 30, 2023. The copyright holder for this preprint (which was not certified by peer review) is the author/funder, who has granted bioRxiv a license to display the preprint in perpetuity. It is made available under aCC-BY 4.0 International license.

control^{R+E} per culture; a-c, control^{R+E} 45 synapses/3 independent cultures, cQKO^{R+E} 52/3, cQKO^{R+E} + RIM1α 44/3, cQKO^{R+E} + PDZ-C2A-P 49/3, cQKO^{R+E} + Zn-C2B 45/3; d-f, control^{R+E} 49/3, cQKO^{R+E} 52/3, cQKO^{R+E} + RIM1α 44/3, cQKO^{R+E} + PDZ-C2A-P 51/3, cQKO^{R+E} + Zn-C2B 47/3; g-i, control^{R+E} 94/6, cQKO^{R+E} 97/6, cQKO^{R+E} + RIM1α 94/6, cQKO^{R+E} + PDZ-C2A-P 95/6, cQKO^{R+E} + Zn-C2B 91/6; j-l, control^{R+E} 46/3, cQKO^{R+E} 46/3, cQKO^{R+E} + RIM1α 45/3, cQKO^{R+E} + PDZ-C2A-P 47/3, cQKO^{R+E} + Zn-C2B 44/3.

Data are mean ± SEM; $**p < 0.01$, $***p < 0.001$ as determined by Kruskal-Wallis followed by Holm multiple comparisons post hoc tests.

bioRxiv preprint doi: https://doi.org/10.1101/2023.10.27.564439; this version posted October 30, 2023. The copyright holder for this preprint (which was not certified by peer review) is the author/funder, who has granted bioRxiv a license to display the preprint in perpetuity. It is made available under aCC-BY 4.0 International license.

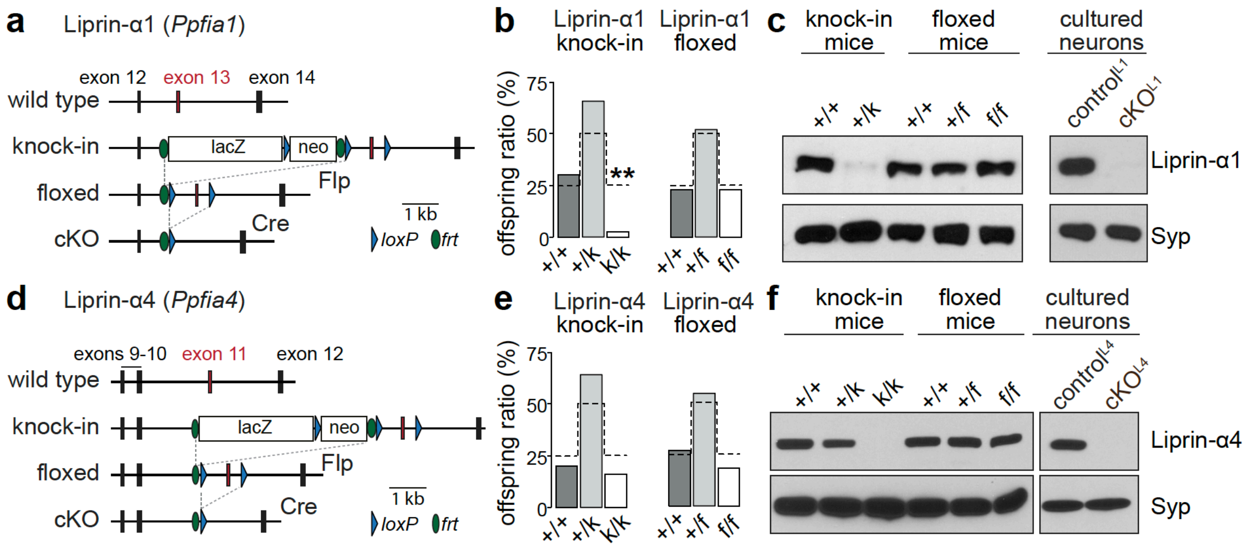

Supplemental figure 3. Liprin-α1 and -α4 mutant alleles

(**a**) Diagram outlining the gene targeting strategy of *Ppfia1* to remove Liprin-α1. The knock-in allele containing loxP sites flanking exon 13 (numbering follows Ensembl ENSMUST00000182226.8) was generated by homologous recombination, chimeric founders were used to establish the knock-in line and subsequently crossed to Flp-transgenic mice[88] to generate the floxed allele.

(**b**) Offspring ratios from Liprin-α1 knock-in (k) or floxed (f) heterozygous breeding pairs. Dotted lines show expected Mendelian ratios; Liprin-α1 knock-in 26 mice/5 litters; Liprin-α1 floxed 35/5.

(**c**) Western blots of whole brain homogenates of wild type, heterozygous and homozygous littermate Liprin-α1 mice before (knock-in) or after Flp recombination (floxed), or of lysates from hippocampal cultures of Liprin-α1 floxed mice infected with a lentivirus expressing Cre (cKO^{L1}) or a recombination deficient truncation of Cre ($control^{L1}$).

(**d-f**) Same as a-c but for *Ppfia4* to remove Liprin-α4, exon 11 is flanked by loxP sites (numbering follows Ensembl ENSMUST00000168515.8); e, Liprin-α4 knock-in 25 mice/4 litters; Liprin-α floxed 44/5.

Data in b and e are shown as observed offspring ratios. **p < 0.01 as determined by Chi-square tests comparing obtained ratios with expected Mendelian ratios.

bioRxiv preprint doi: https://doi.org/10.1101/2023.10.27.564439; this version posted October 30, 2023. The copyright holder for this preprint (which was not certified by peer review) is the author/funder, who has granted bioRxiv a license to display the preprint in perpetuity. It is made available under aCC-BY 4.0 International license.

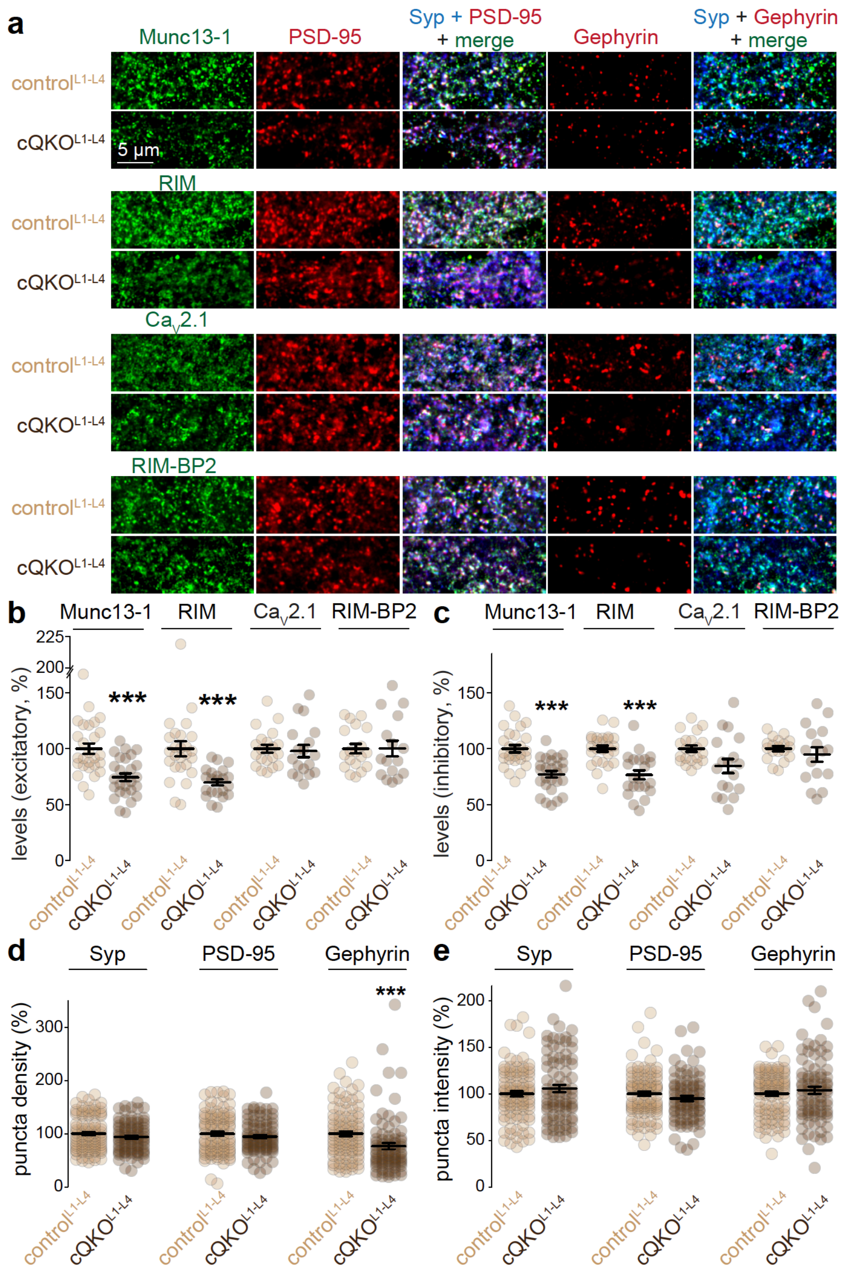

Supplemental figure 4. Confocal microscopic assessment of synaptic protein levels after ablation of Liprin-α1 to -α4

(**a-c**) Example confocal images (a) and quantification of fluorescence intensity levels at excitatory and inhibitory synapses (b, c). Neurons were stained for a protein of interest (Munc13-1, RIM, $Ca_V2.1$ or RIM-BP2), two postsynaptic markers (PSD-95, excitatory, and Gephyrin, inhibitory), and Synaptophysin (Syp). Data are normalized to the average control$^{L1\text{-}L4}$

bioRxiv preprint doi: https://doi.org/10.1101/2023.10.27.564439; this version posted October 30, 2023. The copyright holder for this preprint (which was not certified by peer review) is the author/funder, who has granted bioRxiv a license to display the preprint in perpetuity. It is made available under aCC-BY 4.0 International license.

per culture; Munc13-1, control^{L1-L4} 27 images/3 independent cultures, cQKO^{L1-L4} 27/3; RIM, control^{L1-L4} 26/3, cQKO^{L1-L4} 22/3; Ca$_V$2.1, control^{L1-L4} 21/3, cQKO^{L1-L4} 18/3; RIM-BP2, control^{L1-L4} 19/3, cQKO^{L1-L4} 16/3.

(**d, e**) Quantification of Synaptophysin, PSD-95 and Gephyrin puncta densities (d) and of their fluorescence intensities (e) normalized to the average control^{L1-L4} per culture. The ~20 % reduction in inhibitory synapses is unlikely to fully account for the ~60% decrease in mIPSC frequency and the ~45% decrease in sucrose-evoked responses (Fig. 4w+x and Supplemental fig. 5q+r); control^{L1-L4} 93/3, cQKO^{L1-L4} 83/3.

Data are mean ± SEM; ***$p < 0.001$ compared to cQKO^{L1-L4} as determined by Mann-Whitney U tests in b (RIM and RIM-BP2), c (RIM, RIM-BP2, and Ca$_V$2.1), d, and e, or by Student's t-tests in b (Munc13-1 and Ca$_V$2.1) and c (Munc13-1).

bioRxiv preprint doi: https://doi.org/10.1101/2023.10.27.564439; this version posted October 30, 2023. The copyright holder for this preprint (which was not certified by peer review) is the author/funder, who has granted bioRxiv a license to display the preprint in perpetuity. It is made available under aCC-BY 4.0 International license.

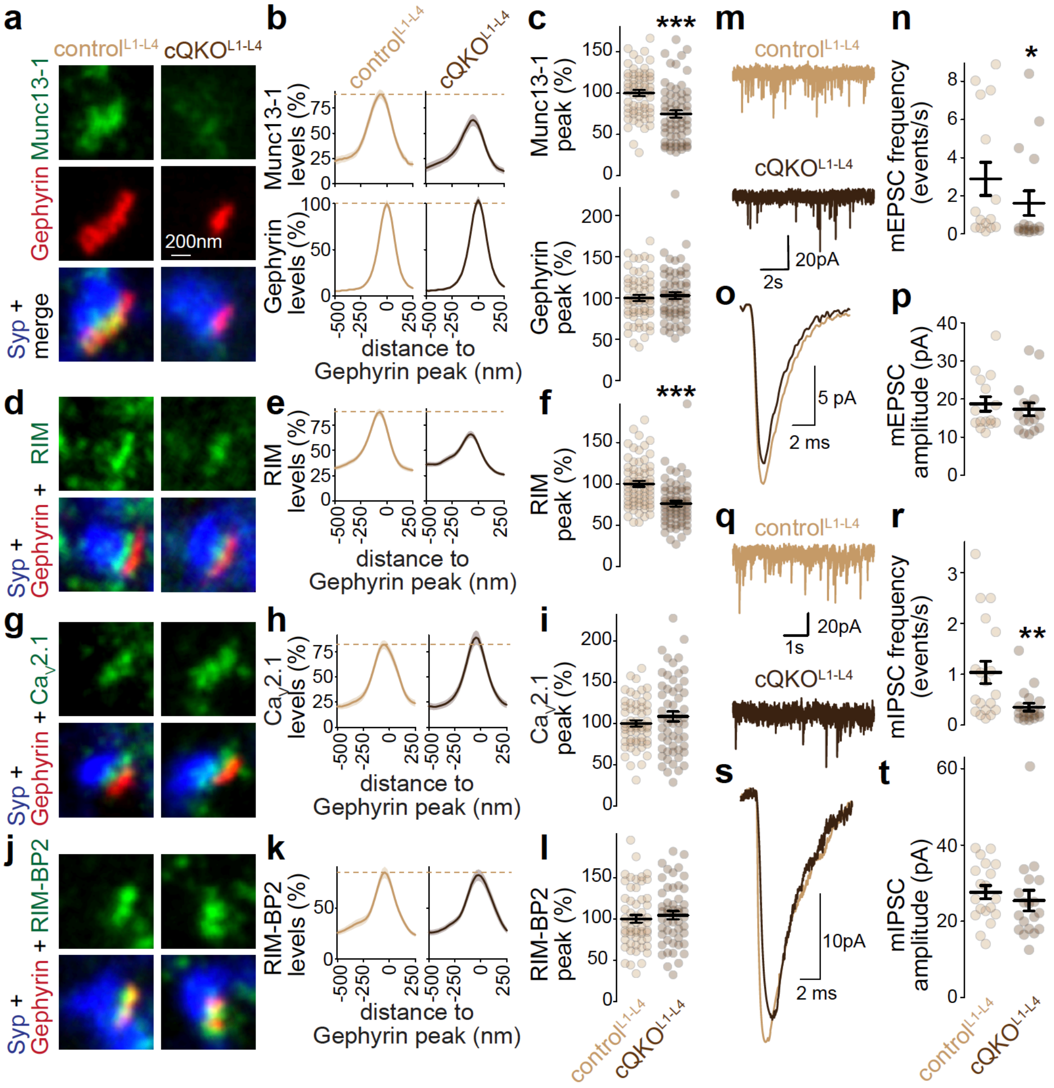

Supplemental figure 5. Assessment of inhibitory synapses by STED microscopy and of spontaneous transmission after Liprin-α ablation

(**a-l**) Example STED images, average line profiles and quantification of the peak intensity of Munc13-1 and Gephyrin (a-c), RIM (d-f), Ca$_V$2.1 (g-i) and RIM-BP2 (j-l) at inhibitory side-view synapses identified by Synaptophysin (Syp) and Gephyrin. Analyses were performed on the experiment shown in Fig. 4c-n as the neurons were co-stained for Gephyrin. Dotted lines in line profile plots mark the levels of control$^{L1\text{-}L4}$, line profiles and peak intensities are normalized to the average control$^{L1\text{-}L4}$ per culture; a-c, control$^{L1\text{-}L4}$ 60 synapses/3 independent cultures, cQKO$^{L1\text{-}L4}$ 61/3; d-f, control$^{L1\text{-}L4}$ 68/3, cQKO$^{L1\text{-}L4}$ 65/3; g-i, control$^{L1\text{-}L4}$ 58/3, cQKO$^{L1\text{-}L4}$ 56/3; j-l, control$^{L1\text{-}L4}$

bioRxiv preprint doi: https://doi.org/10.1101/2023.10.27.564439; this version posted October 30, 2023. The copyright holder for this preprint (which was not certified by peer review) is the author/funder, who has granted bioRxiv a license to display the preprint in perpetuity. It is made available under aCC-BY 4.0 International license.

57/3, cQKO$^{L1\text{-}L4}$ 51/3.

(**m, n**) Example traces (m) of spontaneous miniature excitatory postsynaptic current (mEPSC) recordings and quantification (n) of mEPSC frequency; control$^{L1\text{-}L4}$ 15 cells/3 independent cultures, cQKO$^{L1\text{-}L4}$ 16/3.

(**o, p**) Example traces (o) of an averaged mEPSC from a single cell and quantification of the mEPSC amplitude (p), N as in m+n.

(**q-t**) As in m-p but for mIPSCs; 19/3 each.

Data are mean ± SEM; ***p < 0.001 compared to cQKO$^{L1\text{-}L4}$ as determined by Mann-Whitney U tests (c, f, i, n, p, r and t) or by a Student's t-tests (l).

bioRxiv preprint doi: https://doi.org/10.1101/2023.10.27.564439; this version posted October 30, 2023. The copyright holder for this preprint (which was not certified by peer review) is the author/funder, who has granted bioRxiv a license to display the preprint in perpetuity. It is made available under aCC-BY 4.0 International license.

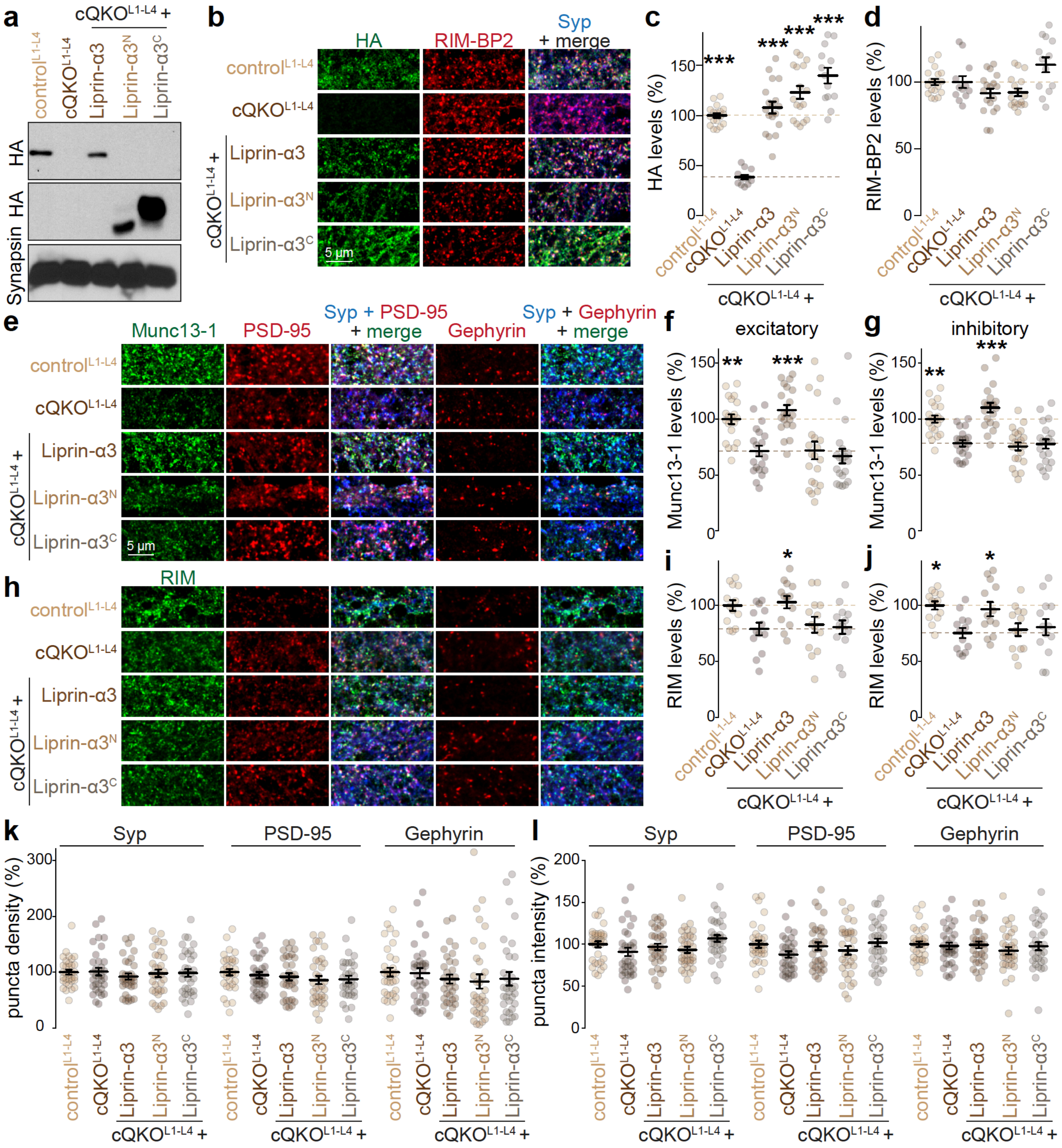

Supplemental figure 6. Assessment of Liprin-α rescue constructs with Western blot and confocal microscopy

(**a**) Western blot to assess expression of Liprin-α3, Liprin-α3^{N} and Liprin-α3^{C}; note that control^{L1-L4} has an HA signal because of the lentiviral expression of HA-tagged Liprin-α3.

(**b-d**) Example confocal images (a) and quantification of fluorescence intensity levels (b+c) of

bioRxiv preprint doi: https://doi.org/10.1101/2023.10.27.564439; this version posted October 30, 2023. The copyright holder for this preprint (which was not certified by peer review) is the author/funder, who has granted bioRxiv a license to display the preprint in perpetuity. It is made available under aCC-BY 4.0 International license.

HA to detect Liprin-α3 and of RIM-BP2 at synapses defined as Synaptophysin (Syp) ROIs. Intensity is normalized to the average control$^{L1\text{-}L4}$ per culture, dotted lines mark the levels of cQKO$^{L1\text{-}L4}$ (dark brown) or control$^{L1\text{-}L4}$ (light brown); control$^{L1\text{-}L4}$ 18 images/5 independent cultures, cQKO$^{L1\text{-}L4}$ 12/5, cQKO$^{L1\text{-}L4}$ + Liprin-α3 18/5, cQKO $^{L1\text{-}L4}$ + Liprin-α3^{N} 17/5, cQKO$^{L1\text{-}L4}$ + Liprin-α3^{C} 13/5.

(**e-j**) Example confocal images and quantification of fluorescence intensity levels at excitatory and inhibitory synapses of Munc13-1 (e-g) and RIM (h-j). Neurons were stained for a protein of interest (Munc13-1 or RIM), postsynaptic markers (PSD-95 and Gephyrin), and Synaptophysin. Data are normalized to the average control$^{L1\text{-}L4}$ per culture, dotted lines mark the levels of cQKO$^{L1\text{-}L4}$ (dark brown) or control$^{L1\text{-}L4}$ (light brown); e-g, control$^{L1\text{-}L4}$ 20/4, cQKO$^{L1\text{-}L4}$ 20/4, cQKO$^{L1\text{-}L4}$ + Liprin-α3 20/4, cQKO $^{L1\text{-}L4}$ + Liprin-α3^{N} 21/4, cQKO$^{L1\text{-}L4}$ + Liprin-α3^{C} 20/4; h-j, 13/3 each.

(**k, l**) Quantification of Synaptophysin, PSD-95 and Gephyrin puncta densities (k) and of their fluorescence intensities (l) normalized to the average control$^{L1\text{-}L4}$ per culture; control$^{L1\text{-}L4}$ 33/4, cQKO$^{L1\text{-}L4}$ 33/4, cQKO$^{L1\text{-}L4}$ + Liprin-α3 33/4, cQKO $^{L1\text{-}L4}$ + Liprin-α3^{N} 34/4, cQKO$^{L1\text{-}L4}$ + Liprin-α3^{C} 33/4.

Data are mean ± SEM; *p < 0.05 **p < 0.01, ***p < 0.00 compared to cQKO$^{L1\text{-}L4}$ as determined by Kruskal-Wallis followed by Holm multiple comparisons post hoc tests in f and k (Gephyrin and Synaptophysin) and l (Synaptophysin), or by one-way ANOVA followed by multiple comparisons Tukey-Kramer post hoc tests in g, i, j, k (PSD-95) and l (PSD-95 and Gephyrin).

bioRxiv preprint doi: https://doi.org/10.1101/2023.10.27.564439; this version posted October 30, 2023. The copyright holder for this preprint (which was not certified by peer review) is the author/funder, who has granted bioRxiv a license to display the preprint in perpetuity. It is made available under aCC-BY 4.0 International license.

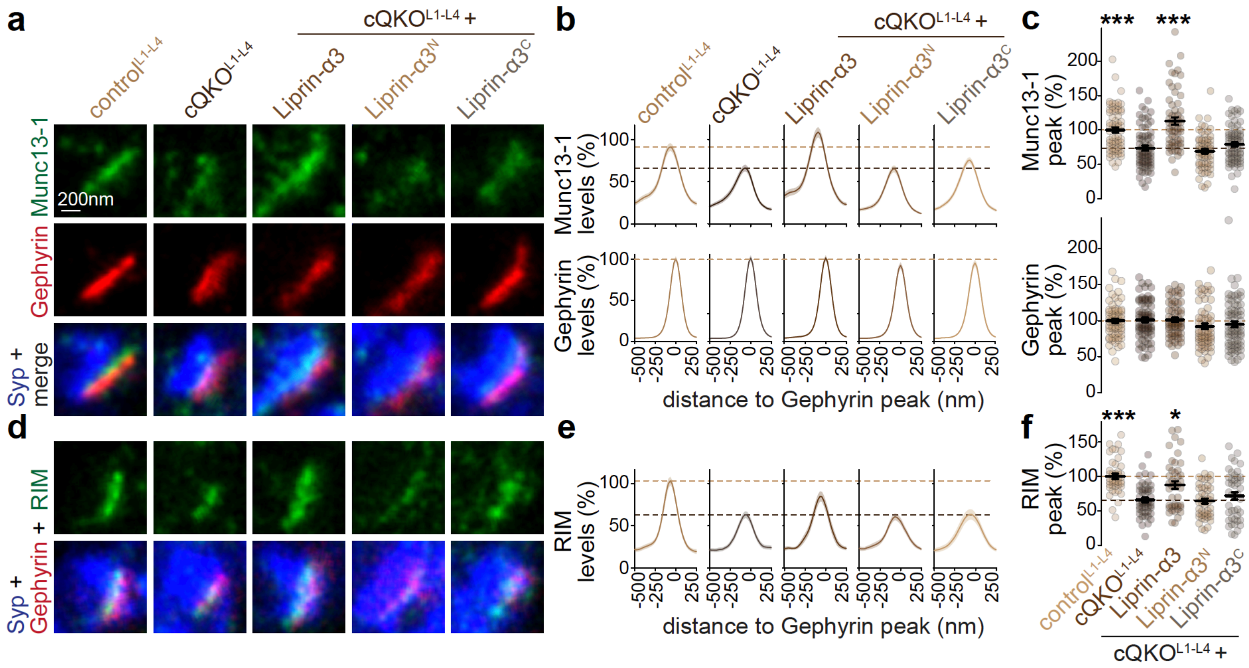

Supplemental figure 7. Assessment of inhibitory synapses by STED microscopy after Liprin-α3 rescue

(**a-f**) Example STED images, average line profiles and quantification of the peak intensity of Munc13-1 and Gephyrin (a-c), and RIM (d-f) at inhibitory side-view synapses identified by Synaptophysin (Syp) and Gephyrin. Analyses were performed on the experiment shown in Fig. 5b-i as the neurons were co-stained for Gephyrin. Dotted lines mark the levels of cQKO^{L1-L4} (dark brown) or control^{L1-L4} (light brown), line profiles and peak intensities are normalized to the average control^{L1-L4} per culture; a-c, control^{L1-L4} 67 synapses/4 independent cultures, cQKO^{L1-L4} 69/4, cQKO^{L1-L4} + Liprin-α3 61/4, cQKO $^{L1-L4}$ + Liprin-α3^{N} 60/4, cQKO^{L1-L4} + Liprin-α3^{C} 74/4; d-f, control^{L1-L4} 45/3, cQKO^{L1-L4} 48/3, cQKO^{L1-L4} + Liprin-α3 43/3, cQKO $^{L1-L4}$ + Liprin-α3^{N} 47/3, cQKO^{L1-L4} + Liprin-α3^{C} 44/4.

Data are mean ± SEM; *p < 0.05, ***p < 0.001 compared to cQKO^{L1-L4} as determined by Kruskal-Wallis followed by Holm multiple comparisons post hoc tests.

bioRxiv preprint doi: https://doi.org/10.1101/2023.10.27.564439; this version posted October 30, 2023. The copyright holder for this preprint (which was not certified by peer review) is the author/funder, who has granted bioRxiv a license to display the preprint in perpetuity. It is made available under aCC-BY 4.0 International license.

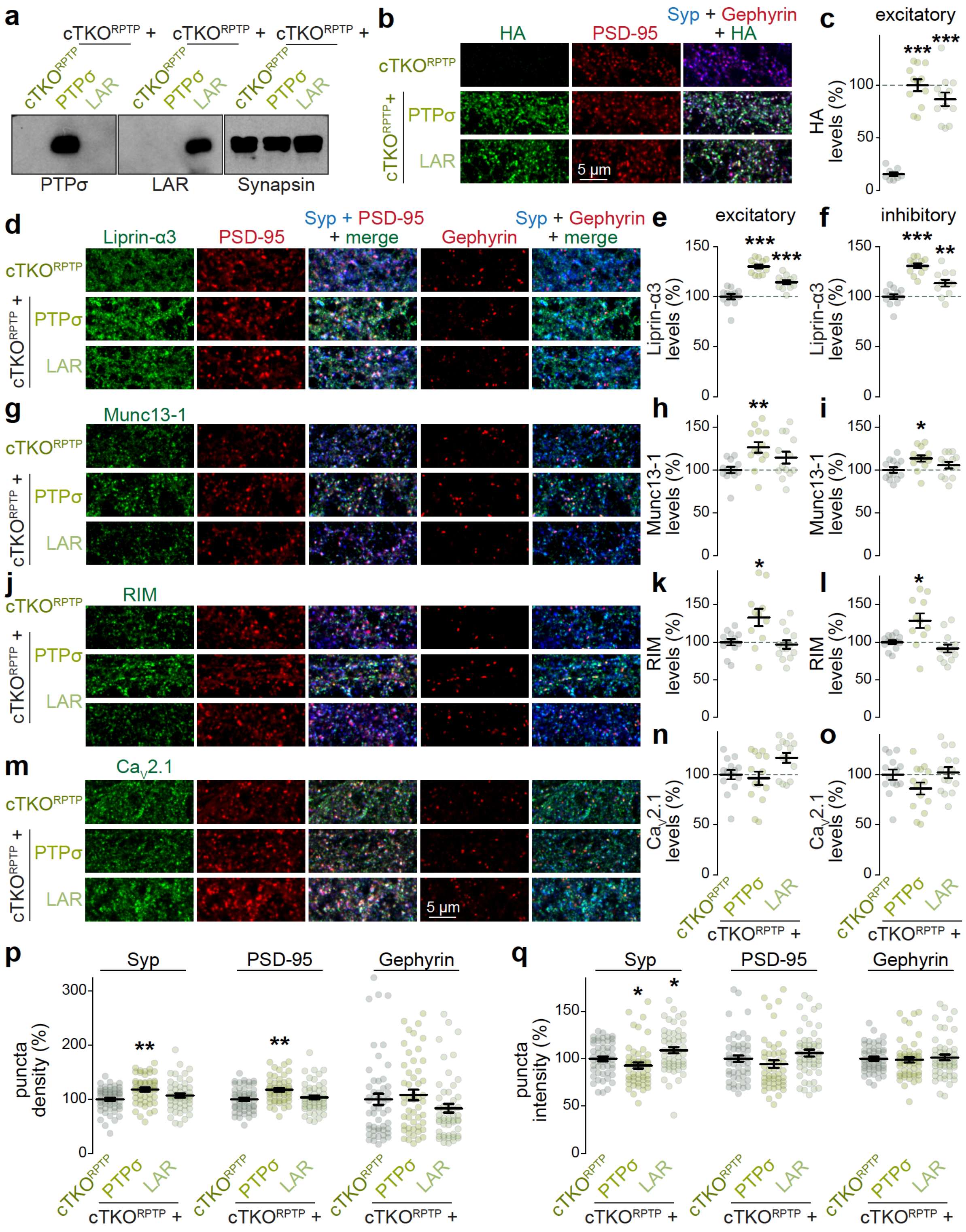

Supplemental figure 8. Assessment of LAR-RPTP proteins with Western blot and confocal microscopy

(**a**) Western blot to assess expression of PTPσ and LAR.

bioRxiv preprint doi: https://doi.org/10.1101/2023.10.27.564439; this version posted October 30, 2023. The copyright holder for this preprint (which was not certified by peer review) is the author/funder, who has granted bioRxiv a license to display the preprint in perpetuity. It is made available under aCC-BY 4.0 International license.

(**b, c**) Example confocal images and quantification of fluorescence intensity levels of HA to detect PTPσ or LAR at excitatory synapses defined as PSD-95 ROIs. Neurons were stained for HA, PSD-95 and Synaptophysin (Syp). Intensity is normalized to the average $cTKO^{RPTP}$ per culture, dotted lines mark the levels of $cTKO^{RPTP}$ + PTPσ; $cTKO^{RPTP}$ 9 images/3 independent cultures, $cTKO^{RPTP}$ + PTPσ 12/3, $cTKO^{RPTP}$ + LAR 12/3.

(**d-o**) Example confocal images and quantification of fluorescence intensity levels at excitatory and inhibitory synapses of Liprin-α3 (d-f), Munc13-1 (g-i), RIM (j-l) and $Ca_V2.1$ (m-o). Neurons were stained for a protein of interest (Liprin-α3, Munc13-1, RIM or $Ca_V2.1$), postsynaptic markers (PSD-95 and Gephyrin), and Synaptophysin. Data are normalized to the average $cTKO^{RPTP}$ per culture, dotted lines mark the levels of $cTKO^{RPTP}$; d-f, 12/3 each; g-i, 13/3 each; j-l, $cTKO^{RPTP}$ 13/3, $cTKO^{RPTP}$ + PTPσ 11/3, $cTKO^{RPTP}$ + LAR 13/3; m-o, 14/3 each.

(p, q) Quantification of Synaptophysin, PSD-95 and Gephyrin puncta densities (p) and of their fluorescence intensities (q) normalized to the average $cTKO^{RPTP}$ per culture. Expression of PTPσ resulted in a mild increase in the number of Synaptophysin and PSD-95 puncta, possibly reflecting a synaptogenic effect, and a mild decrease in the intensity of Synaptophysin; $cTKO^{RPTP}$ 52/3, $cTKO^{RPTP}$ + PTPσ 50/3, $cTKO^{RPTP}$ + LAR 52/3.

Data are mean ± SEM; *$p < 0.05$, **$p < 0.01$, ***$p < 0.001$ compared to $cTKO^{RPTP}$ as determined by Kruskal-Wallis followed by Holm multiple comparisons post hoc tests for c, e, k, l, p (Gephyrin), and q, or by a one-way ANOVA followed by Tukey-Kramer multiple comparisons post hoc tests for f, h, i, n, o and p (Synaptophysin and PSD-95).

bioRxiv preprint doi: https://doi.org/10.1101/2023.10.27.564439; this version posted October 30, 2023. The copyright holder for this preprint (which was not certified by peer review) is the author/funder, who has granted bioRxiv a license to display the preprint in perpetuity. It is made available under aCC-BY 4.0 International license.

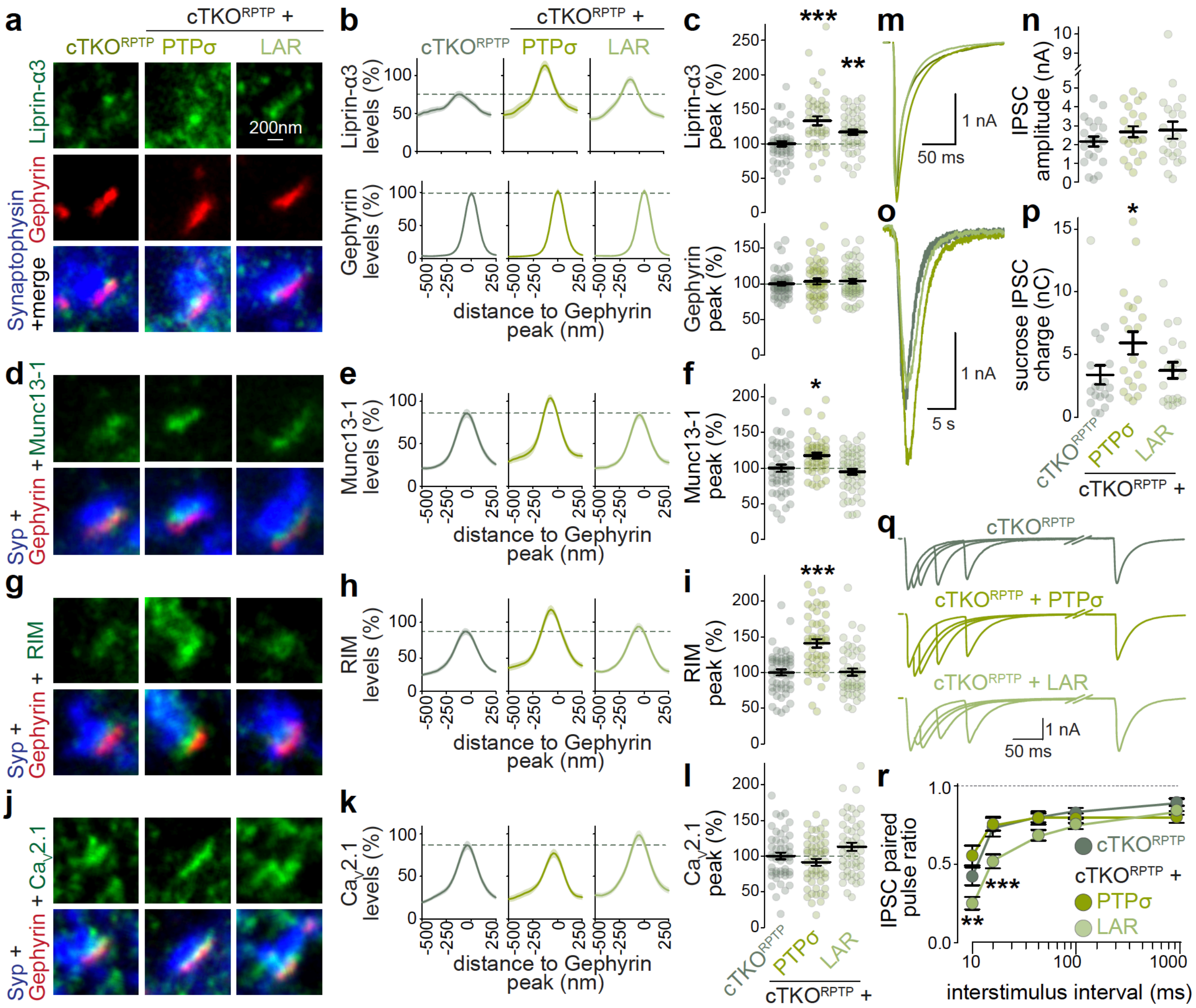

Supplemental figure 9. Assessment of inhibitory synapses by STED microscopy and of synaptic transmission after expression of LAR-PTPs in cTKO^{RPTP} neurons

(**a-l**) Example STED images, average line profiles and quantification of the peak intensity of Liprin-α3 and Gephyrin (a-c), Munc13-1 (d-f), RIM (g-i) and $Ca_V2.1$ (j-l) at inhibitory side-view synapses identified by Synaptophysin (Syp) and Gephyrin. Analyses were performed on the experiment shown in Fig. 7f-q as the neurons were co-stained for Gephyrin. Dotted lines mark the levels of cTKO^{RPTP}, line profiles and peak intensities are normalized to the average cTKO^{RPTP} per culture; a-c, cTKO^{RPTP} 48 synapses/3 independent cultures cTKO^{RPTP} + PTPσ 45/3, cTKO^{RPTP} + LAR 50/3; d-f, cTKO^{RPTP} 49/3, cTKO^{RPTP} + PTPσ 45/3, cTKO^{RPTP} + LAR 50/3;

bioRxiv preprint doi: https://doi.org/10.1101/2023.10.27.564439; this version posted October 30, 2023. The copyright holder for this preprint (which was not certified by peer review) is the author/funder, who has granted bioRxiv a license to display the preprint in perpetuity. It is made available under aCC-BY 4.0 International license.

g-i, $cTKO^{RPTP}$ 51/3, $cTKO^{RPTP}$ + PTPσ 49/3, $cTKO^{RPTP}$ + LAR 47/3; j-l, $cTKO^{RPTP}$ 49/3, $cTKO^{RPTP}$ + PTPσ 51/3, $cTKO^{RPTP}$ + LAR 50/3.

(**m, n**) Example traces (m) and average amplitudes (n) of single action potential-evoked IPSCs. $cTKO^{RPTP}$ 21 cells/3 independent cultures $cTKO^{RPTP}$ + PTPσ 21/3, $cTKO^{RPTP}$ + LAR 22/3.

(**o, p**) Example traces (o) and average GABAR-mediated charge transferred in response to hypertonic sucrose superfusion (p); $cTKO^{RPTP}$ 19/3, $cTKO^{RPTP}$ + PTPσ 21/3, $cTKO^{RPTP}$ + LAR 19/3.

(**q, r**) Example traces (q) and average IPSC paired pulse ratios (r) at increasing interstimulus intervals to estimate P; N as in m+n.

Data are mean ± SEM; *$p < 0.05$, **$p < 0.01$, ***$p < 0.001$ compared to $cTKO^{RPTP}$ as determined by Kruskal-Wallis followed by Holm multiple comparisons post hoc tests (c, f, i, n, and p), by a one-way ANOVA followed by Tukey-Kramer multiple comparisons post hoc tests (l), or by a two-way ANOVA followed by Dunnett multiple comparisons post hoc tests (r).
